# 肺癌患者PICC相关静脉血栓的回顾性分析

**DOI:** 10.3779/j.issn.1009-3419.2015.09.04

**Published:** 2015-09-20

**Authors:** 林 陈, 春华 余, 俊英 李

**Affiliations:** 610041 成都，四川大学华西医院肿瘤中心 Cancer Center, West China Hospital, Sichuan University, Chengdu 610041, China

**Keywords:** 肺肿瘤, 经外周置入中心静脉导管, 静脉血栓, Lung neoplasms, Peripherally inserted central catheters, Vein thrombosis

## Abstract

**背景与目的:**

经外周置入中心静脉导管（peripherally inserted central catheter, PICC）相关静脉血栓会给患者带来极大危害和经济负担，是不容忽视的PICC相关并发症。本研究旨在探讨肺癌患者置入PICC发生静脉血栓的相关因素，以降低PICC相关静脉血栓的发生率，延长导管使用时间。

**方法:**

将2010年1月-2013年9月间住院化疗安置PICC的1, 538例肺癌患者纳为研究对象，对发生PICC相关静脉血栓的患者进行回顾性分析，分析患者年龄、性别、置管静脉、置管肢体、血小板计数、凝血酶原时间（prothrombin time, PT）、纤维蛋白原（fibrinogen, FIB）值与发生静脉血栓有无相关性。

**结果:**

在1, 538例置管患者中，发生静脉血栓38例，发生率为2.47%。患者的年龄、置管肢体、血小板计数、PT对PICC相关性静脉血栓的发生的影响差异无统计学意义（*P* > 0.05），性别（OR=2.194, *P*=0.024）、置管静脉（OR=1.955, *P*=0.006）、FIB值（OR=2.055, *P*=0.028）对PICC相关性静脉血栓的发生的影响差异有统计学意义；女性患者发生率高于男性患者，头静脉置管的发生率高于肘正中静脉、贵要静脉置管，FIB > 4 g/L的患者发生率高于FIB≤4 g/L的患者。

**结论:**

患者性别、置管静脉、FIB值与PICC相关静脉血栓的发生密切相关。在PICC护理工作中，应详细评估患者的情况，进行个体化护理，从而有效降低PICC相关静脉血栓发生率，延长导管使用时间。

## Abstract (en)

**Background and objective:**

It has been proven that peripherally inserted central catheter-related vein thrombosis is a unignorable complication which causes serious harm and economic burden to patients. We designed a study to analyze factors causing peripherally inserted central catheter (PICC)-related vein thrombosis, and find some nursing interventions to reduce the incidence of PICC-related vein thrombosis, and prolong the service time of peripherally inserted central catheters.

**Methods:**

We designed a retrospective analysis. The study participants were 1, 538 lung cancer patients who underwent PICCs placement between January 2010 and September 2013. And tried to determine age, gender, indwelling vein, platelet count, prothrombin time, fibrinogen associated with PICC-related vein thrombosis.

**Results:**

Of the 1, 538 unique PICC placements, 38 patients developed PICC-related vein thrombosis, the incidence was 2.47%. The gender (OR=2.194, *P*=0.024), indwelling vein (OR=1.955, *P*=0.006), fibrinogen (OR=2.055, *P*=0.028) can significantly affect the occurrence of PICC-related vein thrombosis.

**Conclusion:**

Patients' gender, indwelling vein, fibrinogen can affect the occurrence of PICC-related vein thrombosis. Assessing the patients' condition carefully, implementing individual nursing care can reduce the incidence of PICC-related vein thrombosis, and prolong the service time of PICCs.

经外周置入中心静脉导管（peripherally inserted central catheter, PICC）因其操作简单、安全、留置时间长，适用于化疗和静脉营养支持等优点，已广泛应用于临床^[1, 2]^。据报道，PICC致静脉血栓发生率为2.0%-37.5%^[3]^，缩短PICC的留置时间，给患者带来极大危害和经济负担，是不容忽视的并发症。为了解肺癌患者发生PICC相关静脉血栓的相关因素及特点，以减少静脉血栓的发生，并提出个体化的护理措施。本研究对1, 538例安置PICC的肺癌患者进行回顾性分析，现将发生PICC相关静脉血栓病例情况报告如下。

## 1 对象和方法

### 1.1 研究对象

将2010年1月-2013年9月间在华西医院胸部肿瘤病房住院化疗的1, 538例肺癌患者作为研究对象。征得患者和家属同意，签署《经外周置入中心静脉导管同意书》，1, 538例患者均置入PICC。

### 1.2 材料

患者使用同一厂家的PICC，PICC连接肝素帽，透明敷贴（10 cm×10 cm）及医用胶布固定导管。

### 1.3 操作方法

由培训合格的静脉专科护士对患者及家属进行PICC置入相关注意事项的告知，并评估患者血管状况及凝血功能，选择合适的血管在局部麻醉下行PICC置入，送管动作轻柔，减少血管内膜损伤，全程严格无菌操作。置管成功后患者进行胸部X片，确定PICC尖端位于上腔静脉内。

### 1.4 静脉血管的观察与确诊

患者PICC留置期间，严密观察置管肢体有无肿胀、疼痛、臂围增加等临床症状，对有临床症状疑似发生静脉血栓的患者，行彩色多普勒超声以确诊是否发生静脉血栓。

### 1.5 静脉血栓处理方法

确诊静脉血栓的患者遵医嘱进行抗凝治疗，嘱患者抬高患肢，指导患者患肢的活动，禁忌按摩患肢，定期监测凝血功能情况。静脉血栓的治疗方案为口服华法令和（或）皮下注射低分子肝素，抗凝治疗2周左右，再拔除PICC。

### 1.6 统计学方法

采用SPSS 19.0统计软件对数据进行分析，用百分比描述患者血栓在性别、年龄、置管肢体、穿刺静脉、血小板、凝血酶原时间（prothrombin time, PT）、纤维蛋白原值（fibrinogen, FIB）影响下的发生情况；静脉血栓发生的单因素分析采卡方检验，多因素分析采用二元*Logistic*回归分析，以*P* < 0.05为差异有统计学意义。

## 2 结果

### 2.1 一般情况

1, 538例患者，男性1, 113例，女性425例；年龄25岁-84岁，平均年龄为（55.81±10.45）岁，年龄≥60岁为952例， < 60岁为586例；左侧置管322例，右侧置管1, 216例；经贵要静脉置管1, 348例，肘正中静脉置管119例，头静脉置管71例；血小板计数 < 100×10^9^/L的82例，（100-300）×10^9^/L的1, 277例， > 300×10^9^/L的179例；PT < 10 s的97例，10 s-14 s的1, 426例， > 14 s的15例；FIB值< 2 g/L的59例，2 g/L-4 g/L的899例， > 4 g/L的580例。共发生PICC相关静脉血栓38例，其中男性21例，女性17例；年龄30岁-78岁，平均年龄为56岁，年龄≥60岁为23例， < 60岁为15例；经左侧置管13例，右侧置管25例；经贵要静脉置管26例，肘正中静脉置管5例，头静脉置管7例；血小板计数 < 100×10^9^/L的2例，（100-300）×10^9^/L的34例， > 300×10^9^/L的2例；PT < 10 s的3例，10 s-14 s的34例， > 14 s的1例；FIB值< 2 g/L的2例，2 g/L-4 g/L的15例， > 4 g/L的21例。以上患者无一例发生肺栓塞。

### 2.2 静脉血栓的发生率

1, 538例患者中出现置管肢体疼痛、肿胀不适或臂围增加2 cm以上等症状共47例，经彩色多普勒超声检查确诊为静脉血栓的患者共38例，PICC相关性静脉血栓的发生率2.47%。其中2010年共置管341例，发生血栓10例，发生率为2.93%；2011年置管342例，发生血栓7例，发生率为2.05%；2012年置管486例，发生血栓10例，发生率为2.05%；2013年1月-9月共置管369例，发生血栓11例，发生率2.98%。

### 2.3 发生静脉血栓的时间段

38例发生PICC相关静脉血栓的患者中，发生在PICC置管后2周内共28例（71.8%），发生在1个月共内34例（89.5%），发生在置管后1个月以后共4例（10.5%）。最早发生在置管后第2天，最晚发生在置管后的93天。平均天数为（15.12±7.15）天（图 1）。

**1:**
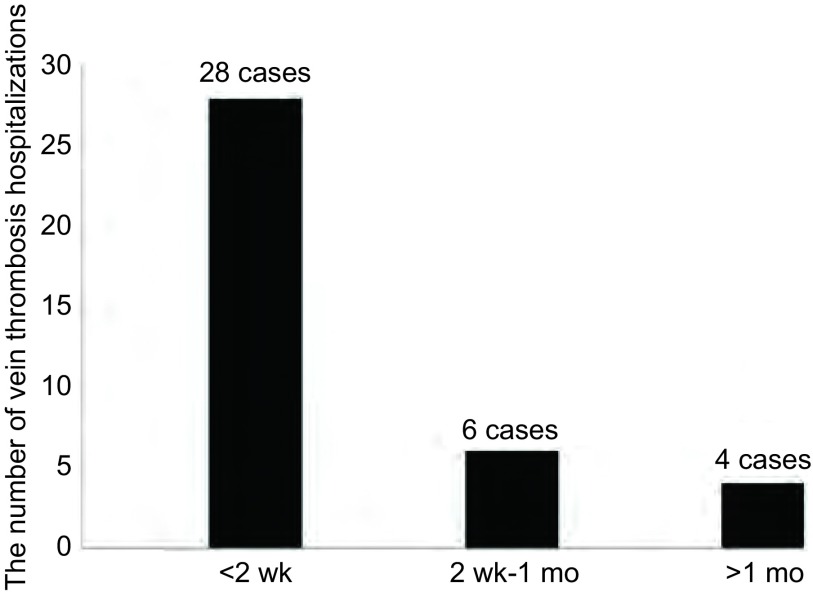
静脉血栓发生时间 The occurrence time of the vein thrombosis

### 2.4 发生静脉血栓相关因素

对1, 538例发生静脉血栓患者相关因素进行分析，单因素分析结果显示，年龄对血栓发生率的影响差异无统计学意义（*P* > 0.05），性别、置管肢体、置管静脉和FIB值差异有统计学意义（*P* < 0.05）（表 1）。

**1:** PICC相关静脉血栓的发生率 The incidence and chi-square test of the vein thrombosis associated with peripherally inserted central catheters

Factor	The cases of patients vein thrombosis		Rate (%)	*P*
	No (*n*=1, 500)	Yes (*n*=38)		
Gender				0.017
Male	1, 092	21	1.88	
Female	408	17	4.00	
Age (year)				0.860
< 60	571	15	2.52	
≥60	929	23	2.42	
Indwelling limb				0.042
Right	1, 191	25	2.05	
Left	309	13	4.03	
Indwelling vein				< 0.001
Basilic vein	1, 322	26	1.93	
Median cubital vein	114	5	4.20	
Cephalic vein	64	7	9.86	
Platelet count (10^9^/L)				0.459
< 100	80	2	2.44	
100-300	1, 243	34	2.66	
> 300	177	2	1.13	
PT (s)				0.523
< 10	94	3	3.09	
10-14	1, 392	34	2.38	
> 14	14	1	6.67	
FIB (g/L)				0.049
< 2	57	2	3.39	
2-4	884	15	1.67	
> 4	559	21	3.62	
PT: prothrombin time; FIB: fibrinogen.				

### 2.5 肺癌患者PICC相关静脉血栓的*Logistic*回归模型

单因素分析结果显示性别、置管肢体、置管静脉及FIB值对PICC相关静脉血栓的发生率的影响有统计学意义（*P* < 0.05），则将性别、置管肢体、置管静脉及FIB值引入二元*Logistic*回归模型，结果显示性别、穿刺静脉、FIB值进入回归模型，且有统计学意义（表 2）。

**2:** *Logistic*回归分析结果 The result of *Logistic* regression analysis

Variable	*β*	SEM	Walds	*P*	Exp (*β*)	95%CI	
						Lower	Upper
Gender	0.786	0.347	5.121	0.024	2.194	1.112	4.252
Vein	0.671	0.242	7.671	0.006	1.955	1.222	3.136
FIB	0.720	0.328	4.812	0.028	2.055	1.070	3.764
Constant	-7.196	2.239	10.334	< 0.001	0.001	-	-

## 3 讨论

PICC相关静脉血栓是后果严重、并受到广泛关注的并发症，发生率为2%-37.5%^[3]^。本研究PICC相关血栓发生率为2.47%。在不同研究静脉血栓的发生率差异较大，这可能与纳入研究的标准有关。有研究^[4]^显示PICC相关血栓发生率为38.5%的研究中，包括了无症状的PICC相关静脉血栓；而本研究主要针对有症状的患者进行分析，发生率为2.47%，这与Evans^[5]^和King^[6]^结果相似。在本研究中，静脉血栓的诊断是采用彩色多普勒超声，而静脉血栓诊断的金标准是静脉造影，但因其成本高，而且有创，不作为首选。

本研究中的38例PICC相关静脉血栓患者，发现血栓的平均天数为（15.12±7.15）天。在Yi等^[7]^和Ong等^[8]^的研究报道中显示PICC相关血栓发生的平均时间分别为（12.45±6.17）天、（12.41±11.0）天，与本研究结果接近，所以在置入PICC 2周左右应警惕静脉血栓的发生。在PICC置入早期，置管过程引起的血管内膜损伤尚未恢复，同时导管留置过程中随着患者的活动也会对血管内膜产生刺激，进而增加血栓形成的风险。所以应对早期带管患者进行重点关注和健康教育：指导患者在置管后72 h做握拳活动，热敷置管静脉以及加强饮水。

研究结果显示，性别是PICC相关静脉血栓的危险因素，女性患者较男性患者更容易发生静脉血栓。而针对此因素的研究国内外观点尚不统一。Allen等^[9]^与母斐等^[10]^的研究提示性别与静脉血栓无明确相关性；而Fletcher等^[11]^研究结果显示男性患者的发生率高于女性患者。但值得注意的是，在本研究中的425例女性患者中，超过85%的患者年龄高于50岁，处于此年龄阶段的女性由于雌激素下降，会增加血液粘度。所以，女性患者也应成为关注的重点人群。

置管静脉的选择与PICC相关静脉血栓的发生有相关性，且头静脉的血栓发生率高于贵要静脉及肘正中静脉。这与Marnejon等^[12]^的研究结果相同。所以在PICC置入前，护理人员应掌握血管结构，充分评估患者血管情况，选择合适的穿刺血管。因贵要静脉管径粗、静脉瓣少，是PICC置入的首选静脉，头静脉内静脉瓣膜较多，其管腔由下至上逐渐变细，分支多，且汇入中心静脉的角度小，增加置管难度及反复送管的次数。同时血管损伤是PICC相关静脉血栓形成的始动因素，置管困难、反复送管会加重对血管的损伤，增加静脉血栓的危险。所以在PICC置入时，尽量避免选择头静脉，同时争取一次性穿刺成功及送管。

此外，研究结果显示FIB值与PICC相关静脉血栓密切相关，FIB值> 4 g/L的患者的静脉血栓发生率高于FIB值≤4 g/L的患者。1946年，Angele等^[13]^提出静脉壁损伤、血流缓慢和血液高凝状态是造成静脉血栓形成的三大要素。纤维蛋白原与全血粘度、血沉及血小板聚集之间呈正相关，提示血浆纤维蛋白原含量升高，可使血液粘度增高，从而使血液处于高凝状态，促进血栓形成^[14]^。所以在PICC置入前和留置过程中，不能忽视患者凝血功能的监测，FIB值> 4 g/L的患者尽量避免PICC置入，如因治疗的需要安置PICC，应该密切监测患者置管肢体的症状与FIB值，及时发现异常，早期干预，早期处理。

年龄、置管肢体、血小板计数及PT不是PICC相关静脉血栓的危险因素。相关研究^[7, 11]^表明置管肢体、PT对PICC相关血栓发生无影响，与本研究结果一致；目前的针对年龄及血小板计数的相关研究结果尚不统一^[15, 16]^，故有待进一步探讨和分析。

肿瘤本身就是一种血栓形成的潜在因素，肿瘤细胞可以激活凝血因子V，能使血液粘度增高^[17]^，而接受PICC置入的肿瘤患者，穿刺本身会对血管壁造成损伤，均会增加静脉血栓的发生率。为减少PICC相关静脉血栓的发生，静脉专科护士在不断提高穿刺技能同时，还要根据患者情况，选择合适的静脉、凝血功能正常的患者进行PICC置入，加强置管肢体的观察和患者健康教育，以便有效控制静脉血栓的发生，延长PICC的使用时间。
